# Analysis of the clinicopathological characteristics, genetic phenotypes, and prognostic of pure mucinous adenocarcinoma

**DOI:** 10.1002/cam4.2726

**Published:** 2019-11-25

**Authors:** Zhencong Chen, Ming Li, Ke Ma, Guoguo Shang, Jiaqi Liang, Jiacheng Yin, Jizhuang Luo, Cheng Zhan, Yu Shi, Qun Wang

**Affiliations:** ^1^ Department of Thoracic of Zhongshan Hospital Fudan University Shanghai China; ^2^ Department of Pathology of Zhongshan Hospital Fudan University Shanghai China

**Keywords:** clinicopathological features, genetic analysis, nomogram, pure mucinous adenocarcinoma, the surveillance, epidemiology, and end results

## Abstract

**Background:**

Primary pure mucinous adenocarcinoma of the lung (PMA) is a rare subtype. However, correlations between clinicopathological features and genetic phenotypes with survival have not been described comprehensively.

**Methods:**

Pure mucinous adenocarcinoma patient information collected from the Surveillance, Epidemiology, and End Results (SEER) database, the Department of Thoracic Surgery, Zhongshan Hospital, Fudan University (FDZSH), and the Cancer Genome Atlas (TCGA) were extracted, evaluated, and compared with other lung adenocarcinomas (LUAD) patient data. Gene Ontology and Kyoto Encyclopedia of Genes and Genomes pathway analyses were performed to explore the functional importance of underlying molecular changes. Overall survival (OS) was evaluated with the Kaplan‐Meier method. Univariate and multivariate analysis through Cox proportional hazard regression identified risk factors that predicted OS, and the results were used to construct a nomogram to predict OS for PMA patients.

**Results:**

Overall, 3622 patients, 41 patients, and 15 patients with PMA were identified from the SEER, FDZSH, and TCGA databases, respectively. There were 345 differentially expressed genes, 30 differentially mutated genes and 72 differentially methylated genes were identified between PMA and other LUAD samples. In the SEER database, PMA had a better prognosis compared to other LUAD. Compared with patients with other LUAD, patients with PMA exhibited unique clinicopathological features, including fewer grade III/IV tumors, less pleural invasion, more early‐stage cancer, and more lower lobe carcinomas. Multivariate analyses showed that age, race, T stage, N stage, surgery, and chemotherapy were independent risk factors. The nomogram had a calibration index of 0.724.

**Conclusions:**

Our research identified unique clinicopathological characteristics and genetic phenotypes for PMA and other LUAD. The nomogram accurately predicted OS.

## Graphical abstract

Clinicopathological characteristics, genetic phenotypes and prognosis factors of primary pure mucinous adenocarcinoma of the lung should be studied in depth. To address this issue, we conducted this large population‐based study. Finally, several clinicopathological features and genetic phenotypes have even been associated with survival in our study. For example, age, race, T stage, N stage, surgery, and chemotherapy were independent risk factors; thyroidtranscription factor‐1 gene and polycomb group ring finger protein 6 gene were relevant to survival.

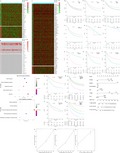

## 1 INTRODUCTION

Lung cancer is the leading cause of cancer‐related death worldwide and is responsible for over 1 700 000 cases every year.^1^, ^2^ Primary mucinous adenocarcinoma of the lung is a rare subtype that includes pure mucinous adenocarcinoma (PMA) and mixed mucinous adenocarcinoma. Pure mucinous adenocarcinoma is defined by the World Health Organization as lung tumor cells comprising goblet and/or columnar cells secreting abundant extracellular mucin and making up more than 50% of the tumor volume; it accounts for only 2%‐5% of all lung adenocarcinomas.^3^, ^4^ Several previous reports have stated that the clinicopathological features and prognosis of PMA are unique from those of other histopathological types of lung adenocarcinoma.^4^ However, most of the previous studies of PMA are limited.

In our study, we reviewed the clinicopathological features, genetic phenotypes, and survival of approximately 4000 PMA patients that we collected from the population‐based the Surveillance, Epidemiology, and End Results (SEER) database, the Cancer Genome Atlas (TCGA) database, and cases treated in our own department. We aimed to better understand the correlations between the clinicopathological features and genetic phenotypes of PMA with survival.

## 2 MATERIALS AND METHODS

### 2.1 Ethical statement

Ethics approval was granted by the Ethics Committee of Zhongshan Hospital, Fudan University (Shanghai, China) (B2018‐106).

### 2.2 Patient selection

Previous studies have suggested that PMA may have clinical features and genomic characteristics distinct from those of other lung adenocarcinoma (LUAD).^4^, ^5^, ^6^ Given that mixed mucinous adenocarcinoma is more complicated and has a lower incidence, mixed mucinous adenocarcinoma was not included in our study.

In our research, patients were selected from the SEER database (2004‐2015), the Department of Thoracic Surgery, Zhongshan Hospital, Fudan University (FDZSH; 2005‐2014), and TCGA. Lung adenocarcinoma was classified according to the 2011 IASLC/ATS/ERS classification system. Patients with an International Classification of Diseases for Oncology, third edition (ICD‐O‐3) histology code of 8480/3 or 8253/3 were included in our study. 

### 2.3 Clinicopathological characteristics

We collected the following information for each patient from the databases: (a) the characteristics of patients (age at diagnosis, sex, and race); (b) features of carcinomas (primary location, tumor size, pathological grade, tumor node metastasis (TNM) stage, and histological subtype); (c) therapy details (record of surgery, radiotherapy, and chemotherapy); (d) follow‐up records (cause of death, cancer‐specific death, and number of months of survival). The TNM stages were classified according to the American Joint Committee on Cancer (AJCC) eighth edition criteria.^7^

### 2.4 Molecular characteristics

The genomic data for the lung adenocarcinoma patients were collected from TCGA and FDZSH. Messenger RNA expression profiles and DNA methylation data (combining level 3 data from Illumina GA and HTSeq platforms) were downloaded from TCGA. DNA variant data were downloaded from TCGA. The mutation status of EGFR, KRAS, ALK, RET, NRAS, ROS‐1, and HER2 was obtained from the pathological reports of patients in Zhongshan Hospital. The status of target genes was obtained from pathologists’ reports and was detected with a fluorescence real‐time polymerase chain reaction kit.^5^

### 2.5 Survival statistical analysis

Survival statistical analyses were performed in IBM SPSS statistics software, version 22.0 (IBM, Inc) and R version 3.5.1 (R Foundation for Statistical Computing). Kaplan‐Meier and log‐rank tests were used to construct and compare survival curves. To confirm whether the upregulated genes or the increased mutational load genes in the PMA cohort were associated with poor survival, we split the patients into a high expression group (>median expression level across all samples) and a low expression group (≤median expression level across all samples); an increased mutational load group (>median mutational level across all samples) and a decreased mutational load group (≤median mutational level across all samples).

Additionally, patient variables whose *P* values were <.1 in univariate analyses were used in multivariate analysis performed with the Cox proportional hazard regression model.^6^ The results of multivariate analysis were used to structure a nomogram and concordance index (c‐index), and calibration plots were used to evaluate model performance. In addition, nomograms were used to compare the predicted survival with the observed survival.^8^ Simultaneously, patients from FDZSH were used as a validation cohort, and the total points for each patient in the validation cohort were calculated according to the established nomogram.

### 2.6 Genome statistical analysis

Genome statistical analyses performed in R version 3.5.1 were as follows: (a) differentially expressed genes (DEGs) among all samples between the PMA and other LUAD patients were analyzed by using the DESeq2 package in R. The statistical threshold for significance was a false discovery rate <0.05 and fold change >2; (b) differentially mutated gene (DMG) analysis was performed between the PMA and other LUAD cohorts, and the frequency of gene mutation in all samples was determined by using the maftools package in R. The difference in the mutation ratio for each gene was identified with Fisher's exact test, and a *P* value < .05 was considered significant; (c) differentially methylated genes (DMeGs) were considered to be genes whose absolute differences in average methylation level between PMA and other LUAD cases were ≥0.15, with *P* < .05 in a Wilcoxon test. In our study, C‐phosphate‐G sites in which methylation was present in at least 80% of the samples were used to evaluate the methylation levels of expressed genes^9^; (d) GO and KEGG analyses were performed with the clusterProfiler package in R to identify the main functions of the DEGs. A significant difference in Gene Ontology (GO) or KEGG pathways was defined as *P* < .05.

## 3 RESULTS

### 3.1 Patient characteristics

Overall, 77 834 patients, 1196 patients, and 514 patients with primary lung adenocarcinoma were identified from population‐based SEER, FDZSH, and TCGA databases; the numbers of PMA patients selected were 3622 (4.65%), 41 (3.43%), and 15 (2.92%), respectively. The baseline clinicopathological characteristics of all participants included in our study are summarized in Table 1.

**Table 1:** Baseline characteristics of primary pure mucinous adenocarcinoma of the lung (PMA) and other lung adenocarcinomas (LUAD) in SEER, FDZSH, and TCGA databases

Characteristics	SEER cohort		FDZSH cohort		TCGA cohort	
	PMA	Other LUAD	PMA	Other LUAD	PMA	Other LUAD
Age (y), median (IQR)	67 (59‐76)	67 (59‐75)	61 (55‐67)	60 (55‐67)	62 (59‐71)	65 (59‐72)
Sex, n (%)						
Female	1958 (54.1)	38 690 (52.1)	24 (65.9)	626 (54.2)	9 (60.0)	267 (53.5)
Male	1664 (45.9)	35 522 (47.9)	17 (34.1)	529 (45.8)	6 (40.0)	232 (46.5)
Race n (%)			**—**	**—**		
White n	2912 (80.4)	57 025 (76.8)			10 (66.7)	386 (77.4)
Black	397 (11.0)	9364 (12.6)			2 (13.3)	51 (10.2)
Others	313 (8.6)	7823 (10.6)			3 (20.0)	62 (12.4)
Grade 3‐4, n (%)	256 (10.9)	18 411 (47.4)	—	—	—	—
Tumor size (cm), median (IQR)	4.2 (2.0‐5.4)	4.0 (2.1‐5.1)	2.2 (1.2‐3.0)	2.9 (1.5‐3.5)	—	—
Primary site, n (%)						
Upper lobe	1338 (36.9)	39 816 (53.7)	16 (39.0)	300 (26.0)	5 (33.3)	301 (60.3)
Middle lobe	173 (4.8)	3379 (4.6)	7 (17.1)	299 (25.9)	1 (6.7)	21 (4.2)
Lower lobe	1654 (45.7)	19 336 (26.0)	18 (43.9)	556 (48.1)	8 (53.3)	164 (32.9)
Others	457 (12.6)	11 681 (15.7)	0 (0.0)	0 (0.0)	1 (6.7)	13 (2.6)
T stage, n (%)						
T1	1012 (27.9)	17 698 (23.9)	34 (90.2)	827 (71.6)	2 (13.3)	168 (33.7)
T2	1233 (34.0)	20 801 (28.0)	7 (9.8)	235 (20.3)	10 (66.7)	266 (53.3)
T3	153 (4.2)	13 760 (18.5)	0 (0.0)	61 (5.3)	2 (13.3)	44 (8.8)
T4	1031 (28.5)	15 756 (21.2)	0 (0.0)	32 (2.8)	1 (6.7)	21 (4.2)
TX	193 (5.4)	6197 (8.4)	0 (0.0)	0 (0.0)	0 (0.0)	0 (0.0)
N stage, n (%)						
N0	2266 (62.6)	29 337 (39.5)	31 (75.6)	846 (73.2)	11(73.3)	323 (64.7)
N1	355 (6.5)	6131 (8.2)	3 (7.3)	102 (8.8)	1 (6.7)	92(18.5)
N2	765 (21.1)	25 047 (33.8)	7 (17.1)	206 (18.0)	2 (13.3)	69 (13.8)
N3	226 (6.2)	10 677 (14.4)	0 (0.0)	0 (0.0)	1 (6.7)	15 (3.0)
NX	130 (3.6)	3020 (4.1)	0 (0.0)	0 (0.0)	0 (0.0)	0 (0.0)
M stage, n (%)						
M0	2221 (61.3)	32 456 (43.7)	41 (100.0)	1150 (99.6)	13 (86.3)	338 (67.7)
M1	1401 (38.7)	41 756 (56.3)	0 (0.0)	5 (0.4)	2 (13.7)	161 (32.3)
AJCC 8th stage, n (%)						
I	1527 (42.2)	16 581 (22.3)	29 (70.7)	769 (66.6)	7 (47.3)	273 (54.7)
II	179 (4.9)	2935 (4.0)	5 (12.2)	150 (13.0)	3(20.0)	116 (23.3)
III	515 (14.2)	16 329 (22.0)	7 (17.1)	226 (19.6)	3 (20.0)	79 (15.8)
IV	1401 (38.7)	38 367 (51.7)	0 (0.0)	10 (0.8)	2 (13.7)	31 (6.2)
Pleural invasion, n (%)					—	—
No/unknown	3379(93.3)	68 073 (91.7)	15 (36.6)	626 (54.2)		
Yes	243 (6.7)	6139 (8.3)	26 (63.4)	529 (45.8)		
Radiation, n (%)						
No/unknown	2765 (76.3)	43 810 (59.0)	40 (97.6)	1141 (98.8)	13 (86.3)	440 (88.2)
Performed	857 (23.7)	30 402 (41.0)	1 (2.4)	14 (1.2)	2 (13.7)	59 (11.8)
Chemotherapy, n (%)						
No/unknown	2157 (59.6)	36 019 (48.5)	25 (41.0)	990 (86.5)	7 (47.3)	171 (34.3)
Performed	1462 (40.4)	38 193 (51.5)	16 (39.0)	155 (13.5)	8 (53.7)	328 (65.7)
Surgery, n (%)			—	—	—	—
No/unknown	1665 (46.0)	54 800 (73.8)				
Performed	1957 (54.0)	19 412 (26.2)				
Surgical method, n (%)					—	—
Lobectomy/bilobectomy	1483 (76.0)	14 885 (77.7)	33 (80.5)	1031 (89.3)		
Partial/wedge/segmental resection	412 (21.1)	3725 (19.4)	7 (17.1)	110 (9.5)		
Pneumonectomy	56 (2.9)	541 (2.9)	1 (2.4)	14 (1.2)		

Abbreviations: FDZSH, the department of thoracic surgery, zhongshan hospital, fudan university; IQR, interquartile range; LUAD, lung adenocarcinoma; PMA, pure mucinous adenocarcinoma; SEER, the surveillance, epidemiology, and end results; TCGA, the cancer genome atlas.

As shown in Table 1, the PMA and other LUAD patients had similar ages; the median ages of the PMA and other LUAD cohorts in the SEER, FDZSH, and TCGA samples were 67, 67, and 61; and 60, 62, and 65 years, respectively. Female patients accounted for the majority of PMA cases. In the SEER cohort, in contrast with the other LUAD cohort, the PMA cohort had fewer grade III/IV tumors and less pleural invasion; consistently, PMA patients tended to undergo surgery (54.0%) and were less likely to have had radiotherapy (23.7%) or chemotherapy (41.4%). However, owing to the limited sample size, the percentage of PMA patients who had accepted chemotherapy or radiotherapy was not similar in FDZSH and TCGA.

Additionally, the most common primary site of PMA was the lower lobe, and the proportion of lower lobe tumors in PMA in the SEER, FDZSH, and TCGA cohorts was 45.7%, 53.3%, and 43.9%, respectively. Moreover, PMA patients tended to have less advanced cases: in the SEER cohort, stage I/II tumors accounted for the largest proportion of PMA (47.1%) patients, yet stage III/IV carcinomas were dominant in other LUAD (73.7%) samples. The FDZSH database showed similar results; however, in the TCGA cohort, the proportion of stage III/IV in PMA (33.7%) was higher than that in other LUAD (22%).

### 3.2 Genetic analysis

**Figure 1:**
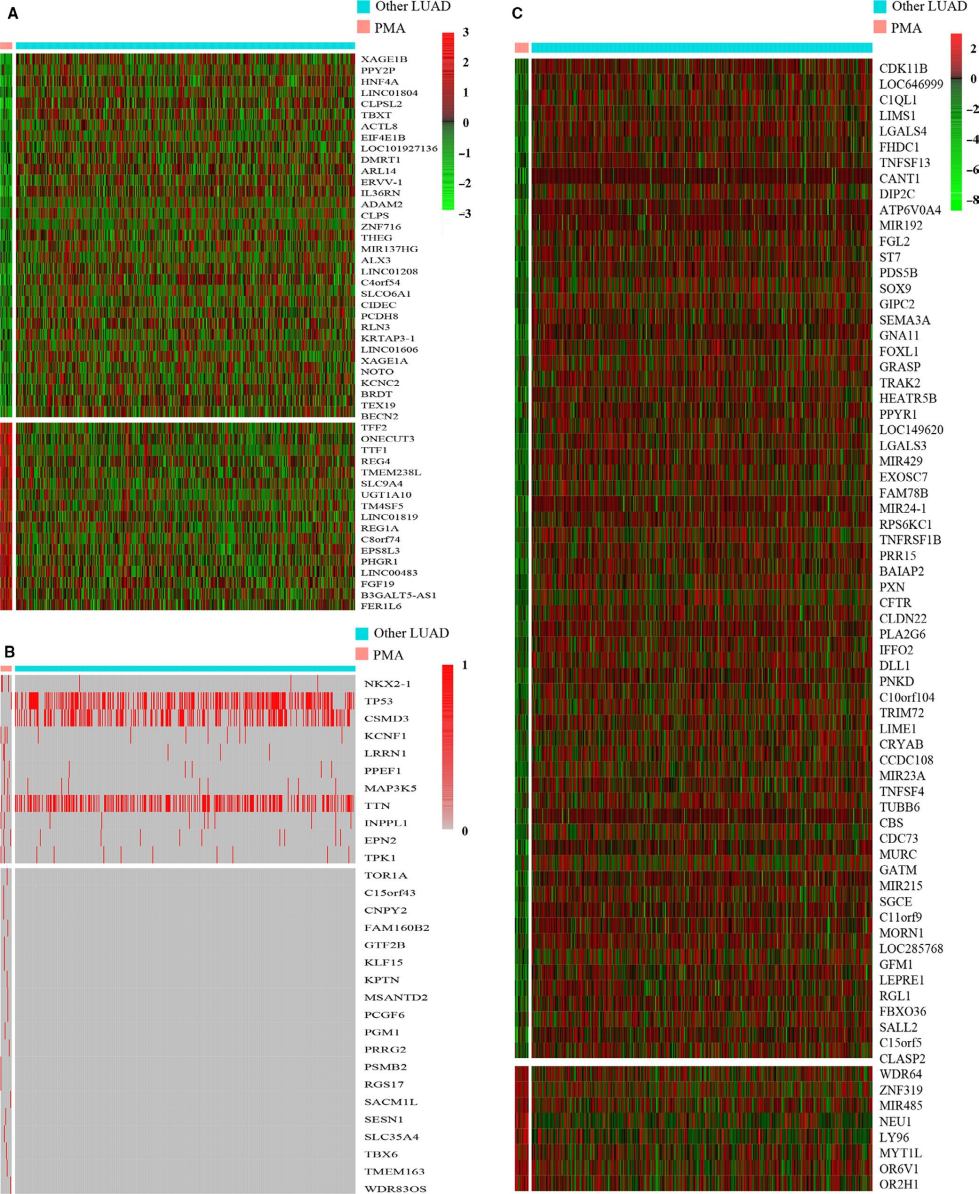
A, Heat map of differentially expressed genes between pure mucinous adenocarcinoma (PMA) and other lung adenocarcinoma (LUAD) subtypes (B) genetic mutation profiles in PMA and other LUAD detected in The Cancer Genome Atlas cohorts (C) heat map of differentially methylated genes between PMA and other lung adenocarcinoma subtypes

In DEG analyses, a total of 514 patients (15 PMA and 499 other LUAD) were included in the TCGA database. The DEGs between the PMA and other LUAD cohorts were selected with strict criteria of fold change >2 and a false discovery rate <0.05. Ultimately, 345 DEGs were identified between PMA and other LUAD, and 24 upregulated genes and 321 downregulated genes in the PMA group were identified. Additionally, a heat map (Figure 1A) was constructed to show the top 50 DEGs between PMA and other LUAD.

A total of 509 patients in TCGA (16 PMA and 493 other LUAD) who had complete mutation data were included in DMG analyses. Thirty DMGs between PMA and other LUAD were selected; the heat map of all DMGs between PMA and other LUAD is shown in Figure 1B. Furthermore, 1196 patients (41 PMA and 1155 other LUAD patients) in FDZSH were included in our study; the mutated genes in PMA and other LUAD are shown in Table 2.

**Table 2:** The mutation status of seven target genes in PMA and other LUAD patients in FDZSH database

Gene	PMA	Other LUAD	*P* value
EGFR, n (%)	6 (14.6)	723 (62.6)	<.001
KRAS, n (%)	18 (43.9)	65 (5.6)	<.001
ALK, n (%)	6 (14.6)	17 (1.5)	<.001
RET, n (%)	2 (4.9)	13 (1.1)	.103
NRAS, n (%)	1 (2.4)	2 (0.2)	.106
ROS‐1, n (%)	0 (0.0)	12 (1.0)	.786
HER2, n (%)	3 (7.3)	79 (6.8)	.543

Abbreviations: LUAD, lung adenocarcinoma; PMA, pure mucinous adenocarcinoma.

We selected 465 patients (18 PMA and 447 other LUAD) with complete methylation data in TCGA and identified 72 DMeGs between the PMA and other LUAD samples. The heat map of all DMeGs is shown in Figure 1C.

**Figure 2:**
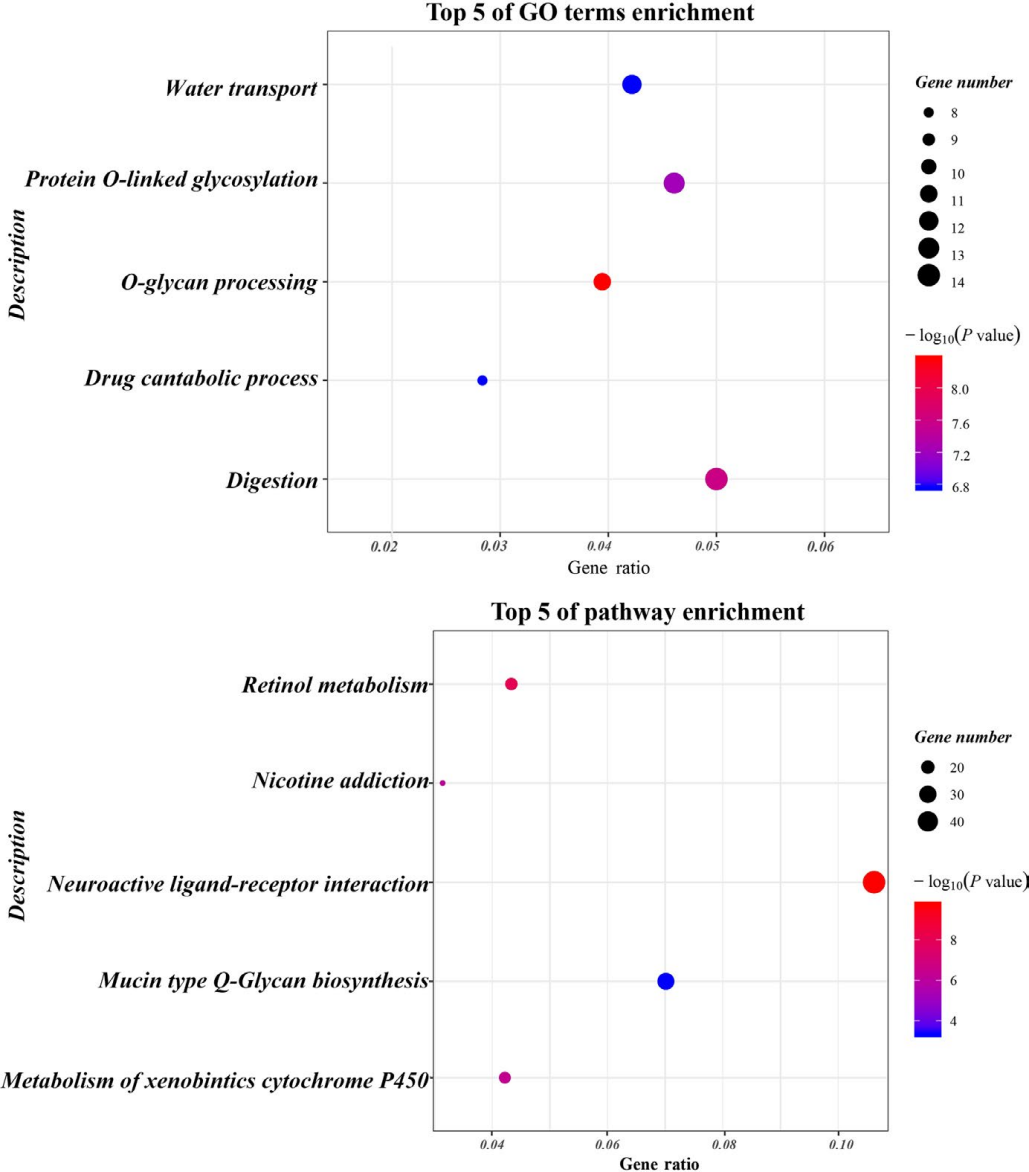
GO and KEGG pathway enrichment analyses on differentially expressed genes between PMA and other LUAD subtypes

In GO and KEGG analyses, there were 59 enriched functional categories for DEGs. The top five significantly enriched GO and KEGG terms of DEGs are shown in Figure 2.

### 3.3 Survival analyses

**Figure 3:**
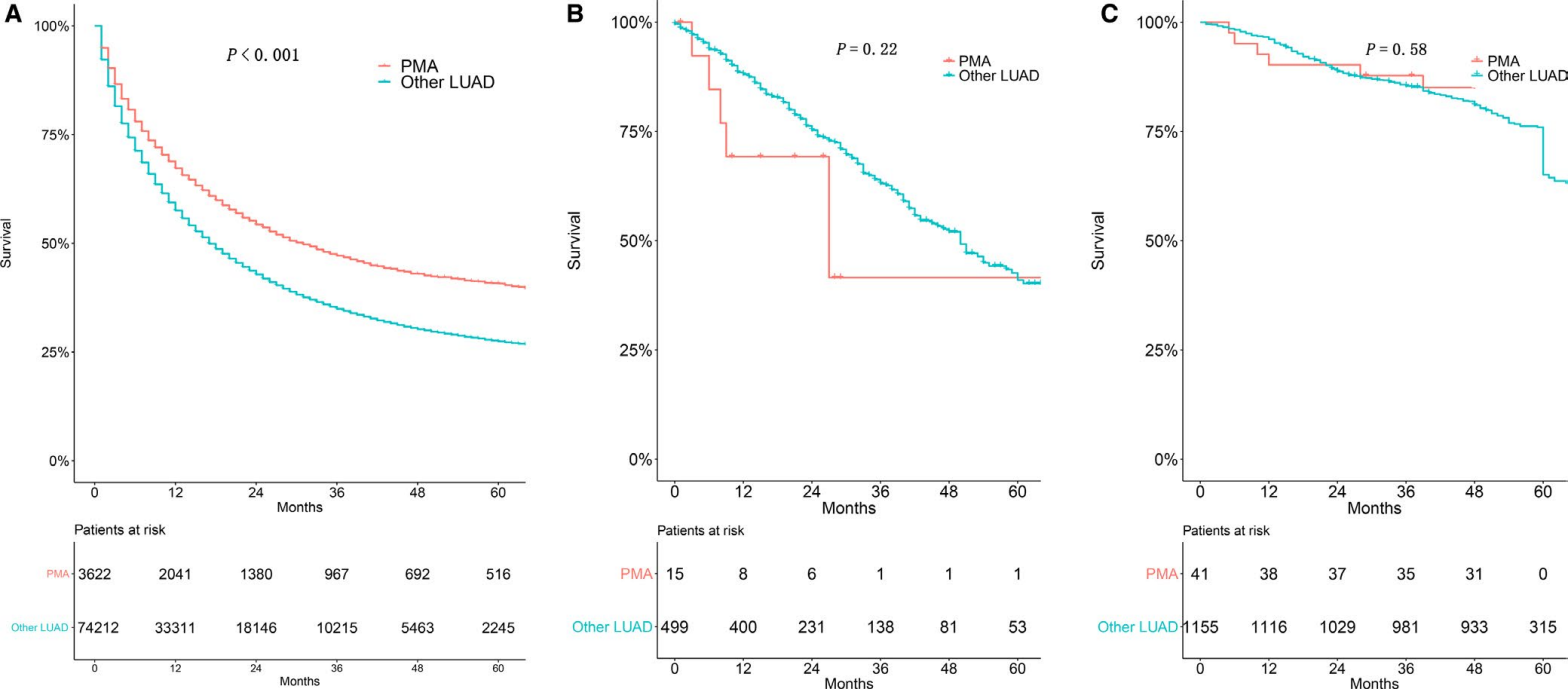
Kaplan‐Meier estimate of overall survival of pure mucinous adenocarcinoma patients and other lung adenocarcinoma patients. (A) SEER cohort, (B) TCGA cohort, (C) FDZSH cohort

The survival outcomes for PMA patients and other LUAD patients are shown in Figure 3. In the SEER cohort, compared with the other LUAD cohort, the PMA cohort had a better prognosis, with a median OS of 31 (95% CI: 27.80‐34.20) months; the 3‐year OS for the PMA and other LUAD cohorts was 47.2% (95% CI: 45.3‐49.0%) and 34.9% (95% CI: 34.5‐35.3%), respectively. Nevertheless, the OS was not significantly different between PMA and other LUAD patients in the FDZSH or TCGA cohort.

In DEGs survival analyses, patients with high expression of cell‐death‐inducing DFF45‐like effector C(CIDEC) (*P* < .001), ADP—xibosylation factor like 14 (ARL14) (*P* =0.003), fibroblast growth factor‐19 (FGF19) (*P* = 0.002), hepatocyte nuclear factor 4 alpha (HNF4A) (*P* = 0.029), and thyroidtranscription Factor‐1 (TTF‐1)  (*P* = 0.032)  were related to poorer prognosis and in DMG survival analyses, patients with Pcgf6 (*P* = .016) had significantly higher hazard ratios.

**Figure 4:**
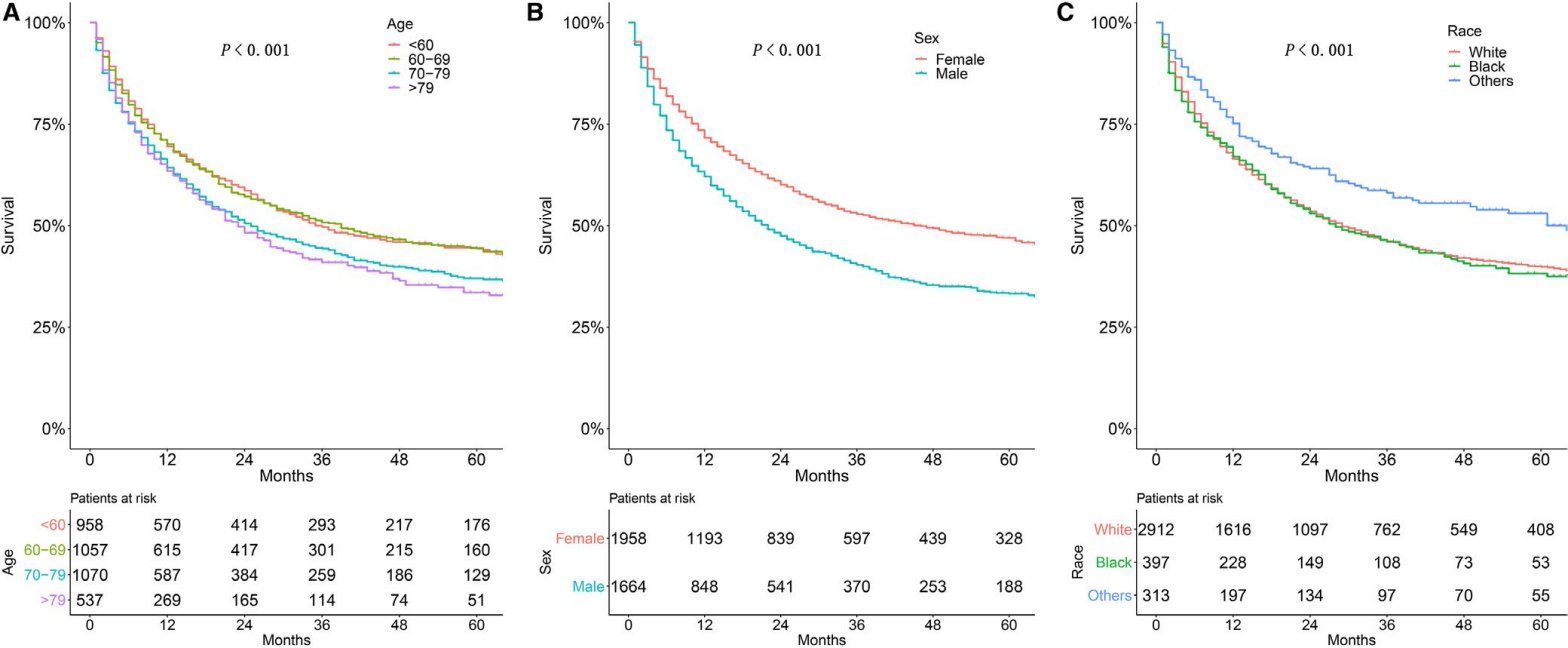
Kaplan‐Meier estimate of overall survival of patients by (A) age, (B) sex, (C) race

**Figure 5:**
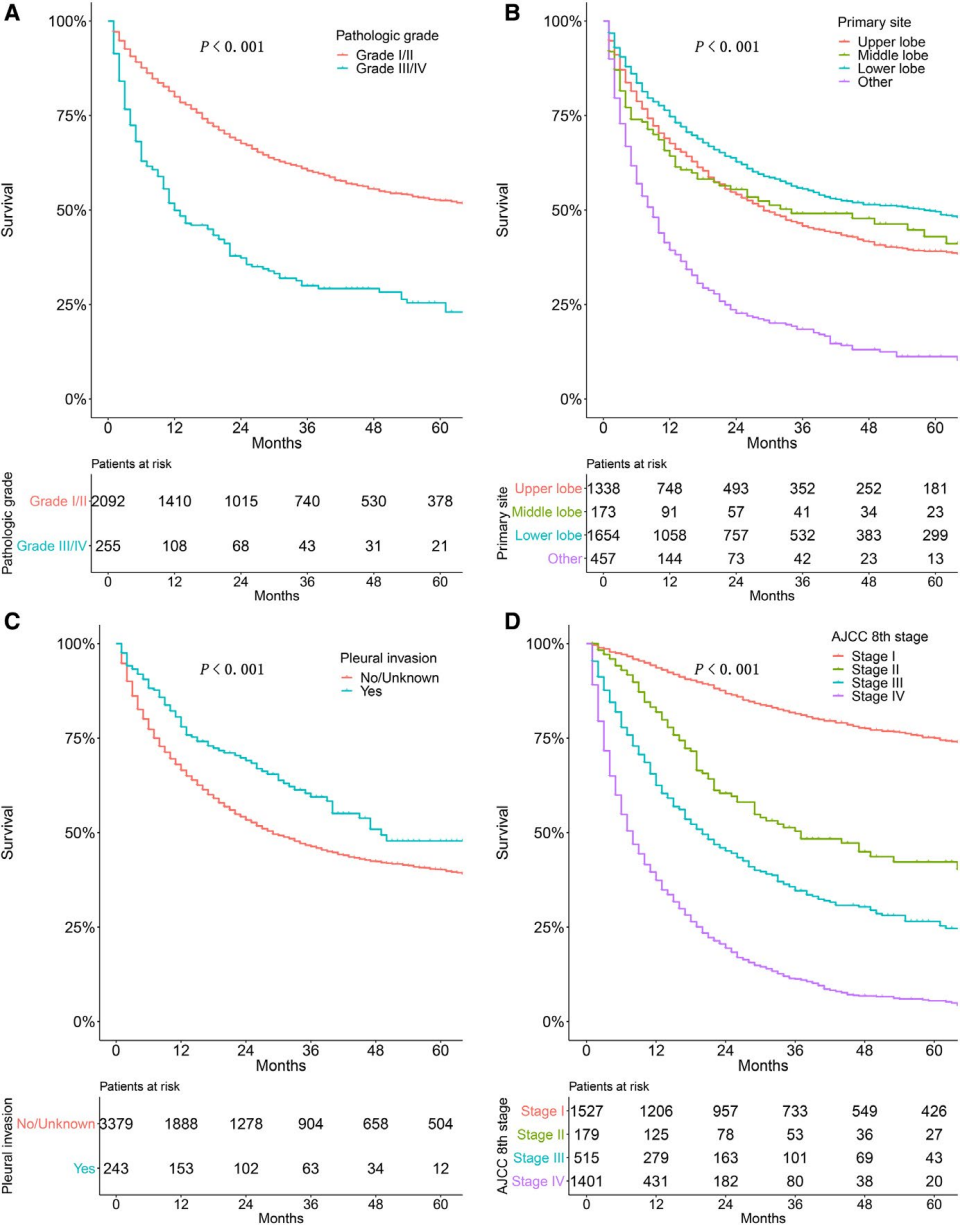
Kaplan‐Meier estimate of overall survival of patients by (A) histological grade, (B) primary site, (C) pleural invasion, and (D) AJCC stage

A total of 3622 PMA patients were included in univariate and multivariate analyses to verify the predictors of survival. As shown in Figure 4, age (*P* < .001; Figure 4A), sex (*P* < .001; Figure 4B), and race (*P* < .001; Figure 4C) were significant prognostic indicators for those patients. As shown in Figure 5, histological grade (*P* < .001; Figure 5A), primary site (*P* < .001; Figure 5B), pleural invasion (*P* < .001; Figure 5C), and AJCC stage (*P* < .001; Figure 5D) were the significant prognostic factors for PMA patients.

**Figure 6:**
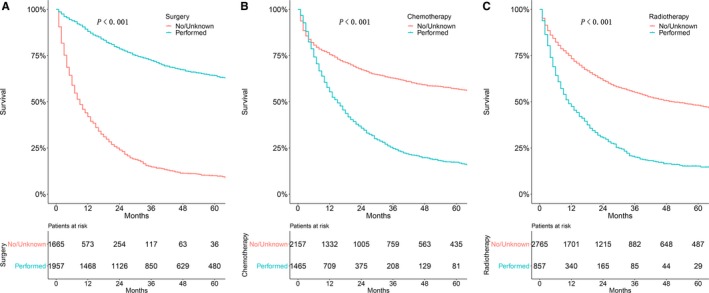
Kaplan‐Meier estimate of overall survival in patients treated by (A) surgery, (B) chemotherapy, or (C) radiotherapy

As shown in Figure 6, patients who had received surgery (*P* < .001; Figure 6A) had significantly better OS than those who did not. However, patients who had not accepted chemotherapy (*P* < .001; Figure 6B) or radiotherapy (*P* < .001; Figure 6C) tended to have a better OS than those who underwent chemotherapy or radiotherapy. Univariate analyses revealed that T stage (*P* < .001), M stage (*P* < .001), and N stage (*P* < .001) all significantly affected OS.

All risk factors with *P* < .1 in the univariate analyses were included in multivariate analyses, except for stage and pleural invasion (because both were associated with T, N, and M stages). Multivariate analyses showed that age (*P* = .021), race (*P* = .003), T stage (*P* = .029), N stage (*P* = .001), surgery (*P* = .002), and chemotherapy (*P* < .001) were able to predict survival of PMA patients. The details of the correlations between survival and factors described above are shown in Table 3.

**Table 3:** Univariate and multivariate Cox proportional hazards analysis

Independent variables	Univariate analysis		Multivariate analysis	
	HR (95% CI)	*P* value	HR (95% CI)	*P* value
Age (y)		<.001		.021
<60		Reference		Reference
60‐69	1.045 (0.946‐1.154)	.389	1.357 (0.994‐1.853)	.055
70‐79	1.249 (1.133‐1.377)	<.001	1.591 (1.186‐2.135)	.002
>79	1.337 (1.188‐1.505)	<.001	1.276 (0.876‐1.860)	.204
Sex		<.001		.159
Female		Reference		Reference
Male	1.381 (1.284‐1.486)	<.001	1.173 (0.940‐1.465)	.159
Race		<.001		.003
White		Reference		Reference
Black	1.098 (0.983‐1.227)	.098	1.185 (0.828‐1.697)	.354
Others	0.711 (0.611‐0.826)	<.001	0.488 (0.312‐0.764)	.002
Grade		<.001		.549
Well/moderately		Reference		Reference
Poorly/undifferentiated	2.362 (2.087‐2.672)	<.001	1.081 (0.839‐1.392)	.549
Primary site		<.001		.720
Upper lobe		Reference		Reference
Middle lobe	0.932 (0.775‐1.120)	.452	1.442 (0.693‐3.001)	.327
Lower lobe	0.764 (0.685‐0.813)	<.001	0.965 (0.712‐1.307)	.816
Others	2.280 (2.066‐2.516)	<.001	1.052 (0.776‐1.427)	.742
T stage		<.001		.029
T1		Reference		Reference
T2	2.082 (1.838‐2.358)	<.001	2.439 (0.937‐6.353)	.068
T3	4.128 (3.420‐4.982)	<.001	2.592 (0.944‐7.114)	.065
T4	5.670 (5.033‐6.389)	<.001	2.617 (1.038‐6.599)	.041
TX	6.228 (5.321‐7.288)	<.001	1.781 (0.691‐4.594)	.232
N stage		<.001		.001
N0		Reference		Reference
N1	2.075 (1.800‐2.392)	<.001	1.318 (0.781‐2.223)	.301
N2	3.607 (3.304‐3.937)	<.001	1.779 (1.341‐2.360)	<.001
N3	4.708 (4.152‐5.338)	<.001	1.729 (1.191‐2.512)	.004
NX	4.964 (4.272‐5.767)	<.001	1.621 (1.118‐2.351)	.011
M stage		<.001		.386
M0		Reference		Reference
M1	5.117 (4.719‐5.549)	<.001	1.125 (0.863‐1.446)	.386
AJCC 8th stage		<.001		
I		Reference		
II	2.954 (2.408‐3.623)	<.001		
III	5.584 (4.888‐6.380)	<.001		
IV	10.768 (9.592‐12.089)	<.001		
Tumor size	1.003 (1.003‐1.004)	<.001	0.888 (0.758‐1.041)	.142
Surgery		<.001		.002
No/unknown		Reference		Reference
Performed	0.170 (0.156‐0.186)	<.001	0.561 (0.388‐0.809)	.002
Radiotherapy		<.001		.654
No/unknown		Reference		Reference
Performed	1.956 (1.809‐2.115)	<.001	1.063 (0.814‐1.387)	.654
Chemotherapy		<.001		<.001
No/unknown		Reference		Reference
Performed	1.705 (1.583‐1.836)	<.001	0.512 (0.407‐0.644)	<.001
Pleural invasion		<.001		.066
Yes		Reference		Reference
No/unknown	1.750 (1.443‐2.124)	<.001	1.969 (0.955‐4.059)	.066

**Figure 7:**
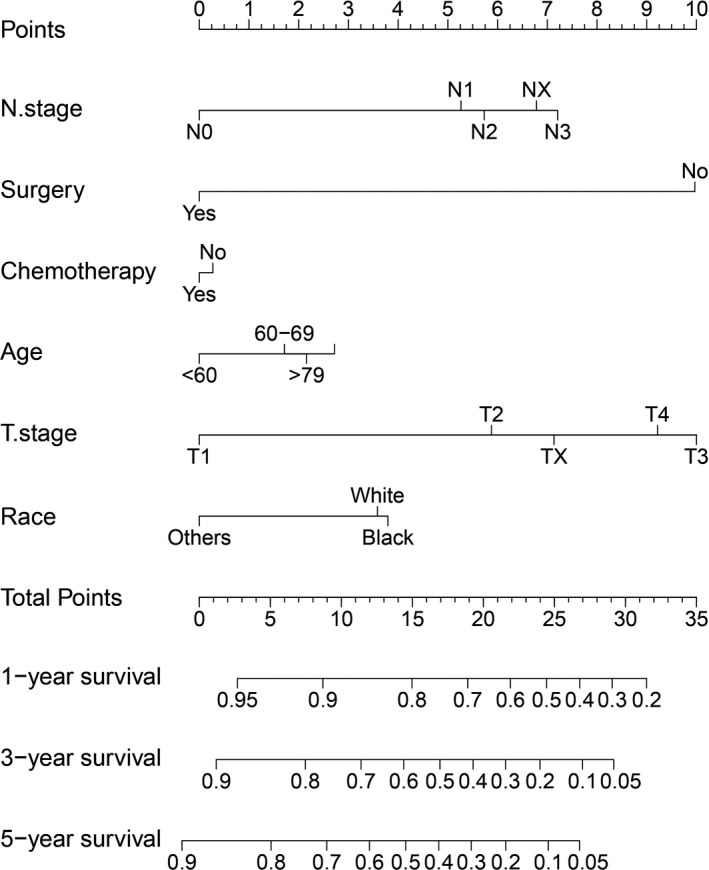
Nomogram to predict 1‐, 3‐, and 5‐year overall survival of patients with pure mucinous adenocarcinoma

**Figure 8:**
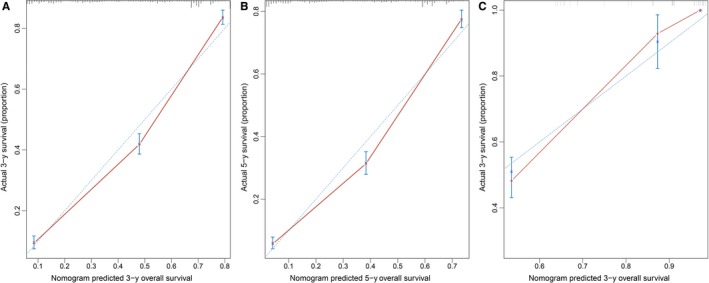
The calibration curve for predicting patient survival at (A) 3 years and (B) 5 years in the primary cohort and at (C) 3 years in the validation cohort. The red line represents equality of the observed and predicted probability

Furthermore, as shown in Figure 7, a nomogram of PMA in the SEER cohort was constructed to compare the predicted survival with the observed survival. The c‐index of this nomogram was 0.724 (95% CI: 0.721‐0.727). The calibration plot of the SEER database and validation cohort is shown in Figure 8. In the validation cohort, the median follow‐up time was 44.2 months (interquartile range, 48‐60 months), and the C‐index of the nomogram for predicting OS was 0.882 (95% CI, 0.812‐0.866), which indicated a good prediction model.

## 4 DISCUSSION

A total of 3622, 41, and 15 patients with PMA were selected from the SEER, FDZSH, and TCGA databases, respectively, to investigate the correlations between clinicopathological features and genetic phenotypes with survival.

Because the SEER cohort had the largest sample size, it was chosen to explore the prognostic indicators of PMA. In the SEER database, in contrast with the other LUAD cohort, PMA patients had less chemotherapy, and more early‐stage tumors and lower lobe tumors; PMA patients tended to accept surgery and had a better prognosis than other LUAD cases. Age, race, T stage, N stage, surgery, and chemotherapy were found to be independently associated with survival in the PMA cohort. A nomogram based on these significant factors was constructed to predict the OS rates of PMA patients. Additionally, patients in the TCGA and FDZSH databases were identified to explore the molecular characteristics of the PMA group. We analyzed the differences in DEGs, DMGs, and DMeGs between PMA and other LUAD samples, then performed GO and KEGG pathway analyses to further determine the functions of these different genes. To the best of our knowledge, our study is the first to comprehensively describe the clinicopathological characteristics as well as the molecular characteristics of PMA.

In our study, the proportion of PMA ranged from 2.92% to 4.65%, a result consistent with findings from previous studies indicating that PMA composes 2%‐10% of lung adenocarcinoma patients.^3^, ^4^ Female patients accounted for the majority of PMA cases and ranged from 53.6% to 58.6% in other studies,^10^, ^11^ thus indicating that PMA is predominant in females. Our research also showed that PMA patients had less pleural invasion and were less likely to be smokers; the most common site for PMA was the lower lobe, a finding consistent with the results of previous studies.^12^, ^13^

PMA is often defined as a malignant tumor, and PMA patients may have poorer prognosis than other LUAD patients.^14^ A previous study has reported that the reason for this finding may because the pressure induced by mucus or the fluid produced by mucinous adenocarcinoma is taken up by the lymphatic system, which may lead to mucinous adenocarcinoma cells becoming more apt to diffuse and transfer and caused a poorer prognosis.^15^ Moreover, Dacic S has suggested that PMA patients tend to not be surgically treated, thus possibly also resulting in a poorer prognosis in PMA patients .^16^ However, in our study, we found that the PMA patients had a better OS compared to other LUAD patients in the SEER cohort, and the OS of PMA was similar between the FDZSH and TCGA cohorts, results partly supported by several previous studies showing that PMA can be classified as a cancer with poor to intermediate prognosis.^17^, ^18^, ^19^ The reasons for these findings may partly be because PMA patients had more early‐stage tumors in the population‐based SEER database. Age, race, T stage, N stage, surgery, and chemotherapy were found to be independent risk factors after multivariate analysis, as shown with a nomogram based on these risk factors. Intriguingly, we also noticed that chemotherapy is negatively associated with survival in univariate analysis but positively associated in multivariate analysis. This may be explained by the fact that application of chemotherapy involves interactions with other factors, including age and stage. Mainly because, in most situations, patients at advanced stage or in older age groups are more likely to undergo chemotherapy. In our nomogram, chemotherapy has a slight but a significant impact on the prognosis in multivariate analysis, and patients who had chemotherapy had a better OS. This result was consistent with findings from a study previously reported by Liu et al^19^ Meanwhile, many studies have reported that EGFR mutations were very rare in PMA and PMA patients may have a very weak response to targeting drugs such as EGFR kinase inhibitors.^20^, ^21^ A study by Liu has reported that other chemotherapy drugs such as pemetrexed may be used in PMA patients and may relieve their symptoms.^19^ As reported in previous research, guidelines for PMA therapy have not been clearly established, and additional research is needed to resolve this important question. Additionally, many studies had demonstrated that pleural invasion was significantly associated with an increased risk of survival.^6^, ^22^, ^23^ However, we observed that pleural invasion is a favorable prognostic factor in univariate analysis in our study; it indicates that pleural invasion has an interaction with another variable, such as AJCC stage and chemotherapy. Therefore, more research is needed to study the role of pleural invasion in survival analyses of PMA in the future.

Moreover, many studies have reported that KARS mutations are common in PMA, but the EGFR mutation rate is relatively low.^24^, ^25^ In our study, we found that EGFR and KRAS were not DMGs, and they were also not associated with OS in the TCGA cohort; however, in the FDZSH database, both EGFR and KRAS were DMGs. The sample size was larger, and the methods used to detect the target genes were more sensitive in the FDZSH cohort, thus potentially leading to the opposite results. Nevertheless, our research showed that only Pcgf6 was both differentially mutated and closely linked to decreased OS. Pcgf6 encodes the Pcgf6 protein, which interacts with some PcG proteins and serves as a transcription repressor.^26^ Previous studies have shown that Pcgf6 is a polycomb group protein that contains a ring finger motif and is a defining component of different PCR1‐type complexes.^27^, ^28^ Zdzieblo et al^29^ have reported that Pcgf6 participates in maintaining embryonic stem cell pluripotency by regulating core pluripotency, mesodermal, and spermatogenesis genes. Lee JH et al^30^ have shown that driver mutations in Pcgf6 enhance cancer cell migration, prompt metastasis, and may act by activating epithelial‐to‐mesenchymal transition. Additional experiments should be performed to explore the therapeutic value of Pcgf6.

Interestingly, we also found that high expression of CIDEC, ARL14, FGF19, HNF4A, and TTF‐1 were related to poorer prognosis. TTF‐1, which belongs to the NKX2‐1 family, is often expressed in the lung, and its expression in lung adenocarcinoma is considered a specific marker of lung adenocarcinoma.^31^, ^32^ As mentioned above, PMA was less likely to harbor EGFR mutations but had a higher frequency rate of KRAS mutations. Consistent with these findings, many studies have reported that TTF‐1 has a strong relationship with EGFR‐mutant lung adenocarcinoma and that TTF‐1 is an oncogene in lung adenocarcinomas with EGFR mutation.^33^, ^34^ Shanzhi et al found that the levels of TTF‐1 expression may guide clinical therapy for LUAD with EGFR mutations.^35^ Moreover, in a previous study, lung adenocarcinoma with KRAS mutation has been found to depend on TTF‐1 expression for growth and to possibly be regulated by survivin protein.^36^ More studies are needed to elucidate the role of TTF‐1 in the process of PMA.

There are some limitations to our study. First, the SEER database did not provide detailed information on aspects such as smoking and alcohol history, and details of chemotherapy or radiation therapy; therefore, we could not analyze those variables in our study. Second, the patients were mostly selected from the USA, and the results might be different  in Asians and Caucasians. Third, the sample size and gene detection method may have caused a lack of association; for example, the numbers of patients in the FDZSH and TCGA cohorts were small, and this might have introduced some bias in the results. Finally, our study was a retrospective study, and large randomized controlled trial studies may be required to confirm the results. Nonetheless, to our knowledge, this is the first report to systematically investigate the relationship between the clinicopathological features and genetic phenotypes of PMA with survival; moreover, patients in our hospital were used to validate the nomogram model and the predictive values of selected genes.

## 5 CONCLUSION

Patients with PMA have unique clinicopathological features compared with other LUAD patients, including more tumors in the lower lobes, early‐stage cancer, and less pleural invasion. In addition, PMA patients had better prognosis than other LUAD patients in the population‐based SEER cohort. Age, race, T stage, N stage, surgery, and chemotherapy were independently associated with OS, and a nomogram model was used to predict the 3‐ and 5‐year OS of PMA patients. Furthermore, our research also assessed the differences in DEGs, DMGs, and DMeGs between PMA and other LUAD; for example, Pcgf6 and TTF‐1. Besides, GO and KEGG analyses were used to explore the associated biological characteristics. Additional validation studies are needed to determine our study's true efficacy.

## CONFLICT OF INTEREST

The authors have no conflict of interest to declare.

## Data Availability

The data that support the findings of this study are openly available in the SEER and TCGA database at https://seer.cancer.gov/seerstat/ and https://portal.gdc.cancer.gov/ and the data which were selected from the Department of Thoracic Surgery, Zhongshan Hospital, Fudan University are not publicly available due to privacy or ethical restrictions
